# Radiation and adjuvant drug-loaded liposomes target glioblastoma stem cells and trigger in-situ immune response

**DOI:** 10.1093/noajnl/vdab076

**Published:** 2021-06-18

**Authors:** Marco Pizzocri, Francesca Re, Elisabetta Stanzani, Beatrice Formicola, Matteo Tamborini, Eliana Lauranzano, Federica Ungaro, Simona Rodighiero, Maura Francolini, Maria Gregori, Alessandro Perin, Francesco DiMeco, Massimo Masserini, Michela Matteoli, Lorena Passoni

**Affiliations:** 1 IRCCS Humanitas Research Hospital, Laboratory of Pharmacology and Brain Pathology, via Manzoni 56, 20089 Rozzano, Milano, Italy; 2 BioNanoMedicine Center NANOMIB, School of Medicine and Surgery, University of Milano-Bicocca, via Raoul Follereau 3, 20854 Vedano al Lambro, Italy; 3 CNR Institute of Neuroscience, Milano, Italy; 4 IRCCS Humanitas Research Hospital, Laboratory of Gastrointestinal Immunopathology, via Manzoni 56, 20089 Rozzano, Milan, Italy; 5 Fondazione Filarete, Milano, Italy; 6 Department of Medical Biotechnology and Translational Medicine, Universita’ degli Studi di Milano, Italy; 7 Department of Neurological Surgery, Fondazione I.R.C.C.S. Istituto Neurologico “C.Besta” Milano, Italy; 8 Department of Pathophysiology and Transplantation, Universita’ degli Studi di Milano, Italy; 9 Department of Neurological Surgery, Johns Hopkins Medical School, Baltimore, Maryland, USA

**Keywords:** blood–brain barrier, drug-loaded liposomes, glioblastoma stem cell, immunogenic cell death, radiotherapy

## Abstract

**Background:**

The radio- and chemo-resistance of glioblastoma stem-like cells (GSCs), together with their innate tumor-initiating aptitude, make this cell population a crucial target for effective therapies. However, targeting GSCs is hardly difficult and complex, due to the presence of the blood–brain barrier (BBB) and the infiltrative nature of GSCs arousing their dispersion within the brain parenchyma.

**Methods:**

Liposomes (LIPs), surface-decorated with an Apolipoprotein E-modified peptide (mApoE) to enable BBB crossing, were loaded with doxorubicin (DOXO), as paradigm of cytotoxic drug triggering immunogenic cell death (ICD). Patient-derived xenografts (PDXs) obtained by GSC intracranial injection were treated with mApoE-DOXO-LIPs alone or concomitantly with radiation.

**Results:**

Our results indicated that mApoE, through the engagement of the low-density lipoprotein receptor (LDLR), promotes mApoE-DOXO-LIPs transcytosis across the BBB and confers target specificity towards GSCs. Irradiation enhanced LDLR expression on both BBB and GSCs, thus further promoting LIP diffusion and specificity. When administered in combination with radiations, mApoE-DOXO-LIPs caused a significant reduction of in vivo tumor growth due to GSC apoptosis. GSC apoptosis prompted microglia/macrophage phagocytic activity, together with the activation of the antigen-presenting machinery crucially required for anti-tumor adaptive immune response.

**Conclusions:**

Our results advocate for radiotherapy and adjuvant administration of drug-loaded, mApoE-targeted nanovectors as an effective strategy to deliver cytotoxic molecules to GSCs at the surgical tumor margins, the forefront of glioblastoma (GBM) recurrence, circumventing BBB hurdles. DOXO encapsulation proved in situ immune response activation within GBM microenvironment.

**Key Points**

- mApoE-liposomes overcome brain barriers and vehicle anti-tumor drugs to glioma cells.
- mApoE-liposomes and concomitant radiotherapy allow therapeutic effects and survival.
- Doxorubicin delivered by mApoE-liposomes causes glioma cell death and immune activation.

**Importance of the Study**

To control tumor growth, and ultimately cure patients, it is essential to develop treatment strategies to kill therapy-refractory cells and to mount robust immunosurveillance to prevent disease recurrence. The GBM stem cell fraction (GSC) present at the tumor edge is the main responsible for therapy resistance and recurrence. Herein, by means of mApoE-functionalized, DOXO-loaded liposomes concomitantly administered with radiation, we provide the proof of concept for a multi-task strategy capable to enhance BBB drug crossing, to cause GSC apoptosis and trigger in situ immune response reactivation. Radiation appear crucially needed to achieve an effective nanovector accumulation and distribution within GBM tumor tissue. The proposed combined approach fulfils the need to selectively target GBM stem cells remaining after surgery within the irradiated field, while preserving a healthy brain by harmful side effects.

Glioblastoma (GBM) is the most common and aggressive primary human brain tumor, associated with very poor prognosis and survival (5-year survival rate less than 5%).^1^ Genetic heterogeneity, angiogenesis, high invasive potential, and stemness properties are crucial factors concurring to GBM recurrence and high mortality rates.^2^ Additionally, the presence of the blood–brain barrier (BBB) in the peripheral areas which host invading cells favors the creation of microenvironmental niches suitable for cancer growth and spreading.^3^

GBM stem-like cells (GSCs) represent a subpopulation of cells characterized by increased resistance to chemo- and radio-therapies. Due to their intrinsic tumor-initiating potential and invasiveness, residual GSCs lead to GBM recurrence and progression. Therefore, GSCs are a relevant target for anti-GBM therapeutic strategies, and GSC-patient-derived xenografts (GSC-PDXs) represent a GBM experimental model close to the clinical scenario.^4^

Thanks to an extensively reprogrammed cellular metabolism, tumor cells survive and proliferate under nutrient and oxygen deprivation. In this context, GBM cells are highly dependent on cholesterol supply for survival^5^ and rely on exogenous cholesterol uptake, mediated by the low-density lipoprotein receptor (LDLR).^6^ Indeed, LDLR is expressed in GBM and its upregulation correlates with tumor progression and drug resistance.^5,7,8^

LDLR expressed at the BBB^9^ is crucially involved in the transport of macromolecules from the bloodstream to the brain, a process referred to as receptor-mediated transcytosis involving receptor-mediated ligand uptake and internalization by endocytosis, transition of the cargo through the cytoplasm, and exocytic release of the cargo.^10,11^ LDLR binds and internalizes Apolipoprotein E (ApoE)-containing lipoproteins that mediate the brain metabolism of cholesterol.^12^

Several nanoscale drug delivery systems involving ApoE-conjugated nanoparticles are currently under investigation as potential vectors to deliver pharmacological agents across the BBB for the treatment of central nervous system disorders.^13^

To date, the nanomedicines approved for cancer treatment are represented by drug-loaded liposomes (LIPs).^14,15^ Thanks to a significantly lower systemic toxicity, liposomal doxorubicin (DOXO) was the first FDA-approved nano-drug in 1995. Since then, many efforts have been made in order to produce more efficient and safe liposomal formulations.^16^ DOXO functions primarily by inhibiting topoisomerase I and II, and intercalating into the DNA double helix to interfere with its uncoiling, ultimately inducing cell death. In addition, cancer cells exposed to DOXO elicit antitumor immunity by immunogenic cell death (ICD), a functionally unique form of cell death that occurs when apoptotic cells secrete distress signals called damage-associated molecular patterns (DAMPs) to trigger an antigen-specific immunity.^17^ A critical step for ICD is the engulfment of dying cells by professional macrophages and the activation of a variety of cells of the innate immunity, essential for the priming of the adaptive immune response.^18,19^ DOXO is effective against numerous solid tumors including GBM. However, DOXO is incapable of BBB crossing.

Here we exploited LIPs conjugated with the ApoE-modified peptide (amino acid residues 141–150) mApoE, previously shown to elicit BBB transcytosis,^20,21^ to vehicle DOXO and treat GSCs-PDXs obtained by intracranial injection of patient-derived GSCs in NOD/SCID mice. Our results provide evidence for therapeutic potential of concomitant radiation/mApoE-DOXO-LIPs administration. Significant tumor growth inhibition, resulting from GSC apoptosis and ICD activation, was associated with increased overall survival upon combined treatment.

## Materials and Methods

### In vitro Transwell BBB Model

Immortalized human cerebral microvascular endothelial cells (hCMECs)^22^ were used as a model of the brain capillary endothelium. hCMEC/D3 cells (7 × 10^4^ cells/cm^2^, p 25–35) were seeded on 12-well transwell inserts coated with type I collagen and cultured with 0.5 ml or 1 ml of culture medium in the upper and in the lower chamber. hCMEC/D3 monolayers integrity was verified by measuring the endothelial permeability (EP) of [^14^C]-sucrose and [^3^H]-propranolol and the transendothelial electrical resistance (TEER) (measured by EVOMX meter, STX2 electrode; World Precision Instruments). For permeability experiments, after 3 h of incubation with 2.5 or 25 µg/ml of DOXO, free or embedded into LIPs, the amount of DOXO in the basolateral compartment was measured by fluorescence and the EP to DOXO calculated as described.^23^ LIP integrity after BBB crossing was evaluated by Nanoparticle Tracking Analysis (NanoSight NS300, Malvern Panalytical). Impact of free-DOXO or DOXO-LIPs on cell monolayers was checked by measuring TEER and EP of Lucifer Yellow (50 μM) after hCMEC/D3 incubation. For cytotoxicity experiments, hCMEC/D3 were cocultured with U87-MG, A172 cells (5.4 × 10^4^ cells/well) adherent to the bottom of the basolateral compartment. 25 µg/ml of DOXO, free or embedded in LIP, were added to apical compartment and incubated for 3 h. Then, upper inserts were removed and GBM cells cultured for an additional 48 h in the lower chambers. Viability was evaluated by MTT assay.

### Patient-derived GSCs

Patient-derived GSC cultures were obtained from GBM patients undergoing surgery for brain tumor removal. Surgical specimens were collected from consenting patients in the Department of Neurosurgery at Neurological Institute “C. Besta” (Italy) under “C. Besta” research ethics committee approval. Tumor samples were processed as by Galli et al.^24^ Neurospheres were subsequently split 1:10 every 7–10 days by mechanical dissociation. All experiments were performed before passage 11.

### GSC Orthotopic Xenografts and Treatments

To study mApoE-DOXO-LIP antitumor activity, luciferase transfected GSC1luc cells (4 × 10^4^) were stereotactically injected into the right striatum (from bregma: x = 2,5 mm; y = 1 mm posterior; z = −3 mm, Digital Model 900 Small Animal Stereotaxic Instrument, Better Hospital Equipment Corp) of 4-week-old male NOD/SCID mice (NOD.CB-17-Prkdcscid/J, Charles-River). Luciferase stable expression was obtained using the pRRL.sin.PPT.CMV.Luciferase.iresEMCVwt.eGFP.pre lentiviral vector kindly provided by Dr. C.Boccaccio (Candiolo Cancer Institute, Torino, Italy).^25^ Treatments started at 8 weeks after tumor injection (D60) when tumors were detectable by BLI imaging (IVIS® II Imaging, Caliper Life Sciences-PerkinElmer). In order to obtain comparable results, animals were randomized according to BLI imagines into groups homogeneous for tumor dimension. All treated mice received 35 μg/dose/mouse of DOXO delivered by DOXO-LIPs or mApoE-DOXO-LIPs administered intraperitoneal. Whole-brain radiation (2Gy) was performed using an X-ray irradiator operating at 12 mA/190 kV (RADGIL, Gilardoni). mApoE-DOXO-LIPs were administered 16–18 h after radiation.

### mApoE-LIP Biodistribution

All procedures involving animals were conducted according to Italian laws and the Animal Utilization Protocols approved by the Italian Ministry of Health. 6–8 weeks old male Balb/c mice (3 mice/group; Charles-River Laboratories) were injected via the tail vein with 100 μl of radiolabelled mApoE-DOXO-LIP (10 mM total lipids, 5 mg/kg DOXO, 0.5 µCi/mouse [^3^H]-Sm, 0.3 µCi/mouse [^14^C]-DOXO) or free DOXO (5 mg/kg, 0.3 µCi/mouse [^14^C]-DOXO) in PBS. After mice were sacrificed a total of 0.1 g of each tissue or 100 μl of blood, in triplicate, were solubilized at 55°C for 2 h and cooled to room temperature. Radioactivity was measured by liquid scintillation counting. Data were expressed as a percentage of injected dose on total organ weight/volume ± SD. The possible radioactivity derived from the blood was subtracted from the radioactivity values measured in the brain (−5% of measured radioactivity).

### Statistical Analysis

In vitro data are expressed as mean values ± standard deviation (SD) or standard error (SE) of at least 3 independent experiments performed in replicates. Student's t-test was used for pairwise comparison. Significance was defined at *P* < .05. In vivo statistical significance among treatments was evaluated by ANOVA using correction post hoc tests (Bonferroni or Turkey's multiple comparisons as indicated). Statistical analyses were performed using Prism 8 (GraphPad Software).

## Results

### mApoE-DOXO-LIP Are Able to Cross the BBB In Vitro and Affect GBM Cell Viability

**Figure 1.**
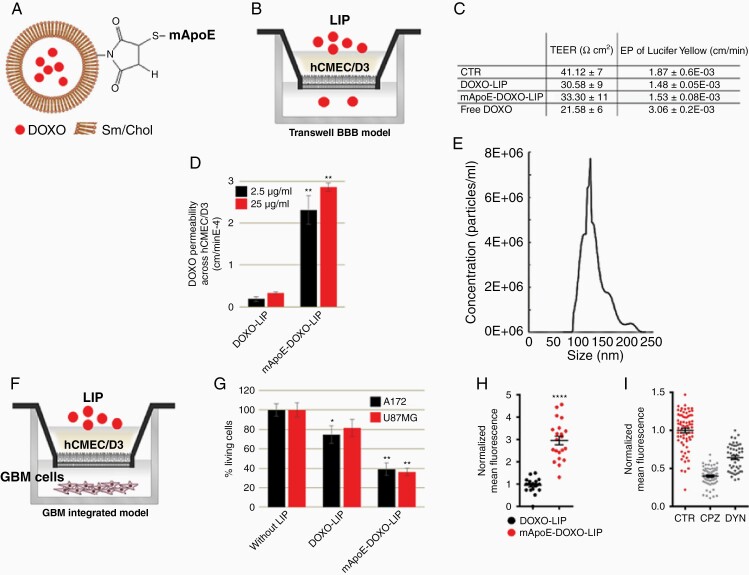
In vitro BBB crossing and GBM cell viability. (A) Schematic representation of Sphingomyelin (Sm)/Cholesterol (Chol) LIPs functionalized with mApoE peptide and embedding DOXO. (B) Schematic representation of the in vitro BBB model prepared using hCMEC/D3 cells seeded on the transwell filter. (C) TEER and EP to LY of endothelial monolayers after 3 h incubation with DOXO (25 μg/ml), free or embedded into LIPs. (D) EP to embedded DOXO determined by adding samples in the apical compartment and monitoring DOXO fluorescence in the basolateral one after 3 h incubation. (E) mApoE-DOXO-LIP size after BBB crossing determined by nanoparticle tracking analysis of LIPs present in the basolateral compartment. (F) Schematic representation of the integrated transwell system prepared seeding adherent GBM cells in the basolateral compartment. (G) hCMEC/D3 cells, in coculture with U87MG or A172, were incubated with DOXO-LIPs or mApoE-DOXO-LIPs added in the apical compartment (DOXO 25 μg/ml). After 3 h incubation, the transwell insert was removed and GBM cells viability was determined by MTT assay after an additional 72 h of culture. (H) Nuclear DOXO quantification in U87MG cells incubated with the indicated LIPs and (I) with mApoE-DOXO-LIPs alone (CTRL) or in the presence of the endocytosis inhibitor chlorpromazine (CPZ) and dynasore (DYN). All data are reported as the mean of at least 3 independent experiments ± SD, *P<.05, **P< .01, ****P < .0001.

LIP (Figure 1A) physiochemical characterization showed that, under physiological conditions (pH 7.4), the final preparations were formed by LIPs < 130 nm, monodispersed, and negatively charged. Release kinetic from mApoE-DOXO-LIP showed a DOXO release rate of 0.11%/h at pH 5 and 0.05%/h at pH 6.5 and 7.4 (Supplementary Figure S1).

mApoE-DOXO-LIP ability to cross the BBB and target GBM cells was investigated in vitro using a transwell system integrated with a human brain capillary endothelial cell monolayer (hCMEC/D3), as a model of BBB (Figure 1B). mApoE-DOXO-LIPs, DOXO-LIPs, or free-DOXO were added to the apical compartment and the barrier integrity was measured after treatment. A higher transendothelial electrical resistance (TEER), together with lower permeability (EP) to Lucifer Yellow (LY), was detected in hCMEC/D3 cell barriers exposed to DOXO encapsulated into LIPs compared to free-DOXO (Figure 1C), indicating that the drug incorporation into LIPs reduces its cytotoxicity. The endothelial permeability to DOXO, either free or incorporated into LIPs, was measured by quantitation of DOXO fluorescence in the basolateral compartment over time. A significant enhancement (approximately 5-fold) in DOXO permeation through the BBB was observed for mApoE-DOXO-LIPs compared to DOXO-LIPs (Figure 1D). Measurement of LIP size after BBB crossing revealed the lack of significant differences in LIP dimensions, confirming the preserved integrity of mApoE-DOXO-LIP (Figure 1E; Supplementary Figure S1C).

To assess if encapsulated DOXO was able to exert anti-tumor activity after BBB crossing, the BBB transwell model was integrated with U87MG and A172 GBM cells growing in the basolateral chamber (Figure 1F) and GBM cell viability was evaluated by MTT assay. Results (Figure 1G) showed that mApoE-DOXO-LIPs, but not DOXO-lip, added in the upper chamber, significantly reduced, after BBB crossing, the viability of both U87MG and A172 cells by approximately 53% and 60%, respectively.

To confirm that mApoE-DOXO-LIPs cytotoxicity was due to specific LIP intracellular uptake, DOXO fluorescence was measured by the analysis of confocal images. Results indicated a 3- to 6-fold increase of nuclear DOXO triggered by mApoE surface LIP functionalization (Figure 1H; Supplementary Figure S2). Incubation with the inhibitor of clathrin-mediated endocytosis, Chlorpromazine (CPZ), or the dynamin inhibitor Dynasore (DYN) significantly reduced mApoE-DOXO-LIP intracellular uptake compatible with a receptor-mediated recognition of the mApoE peptide responsible for the cellular uptake (Figure 1I).

These data provide the in vitro proof-of-concept that, relative to DOXO-LIP, functionalization with mApoE promotes the LIP permeability across the endothelial barrier and induces their clathrin-mediated endocytosis by tumoral cell lines, thus resulting in significant cell death.

### mApoE-DOXO-LIP Cellular Uptake and Cytotoxicity in Patient-derived GSCs

**Figure 2.**
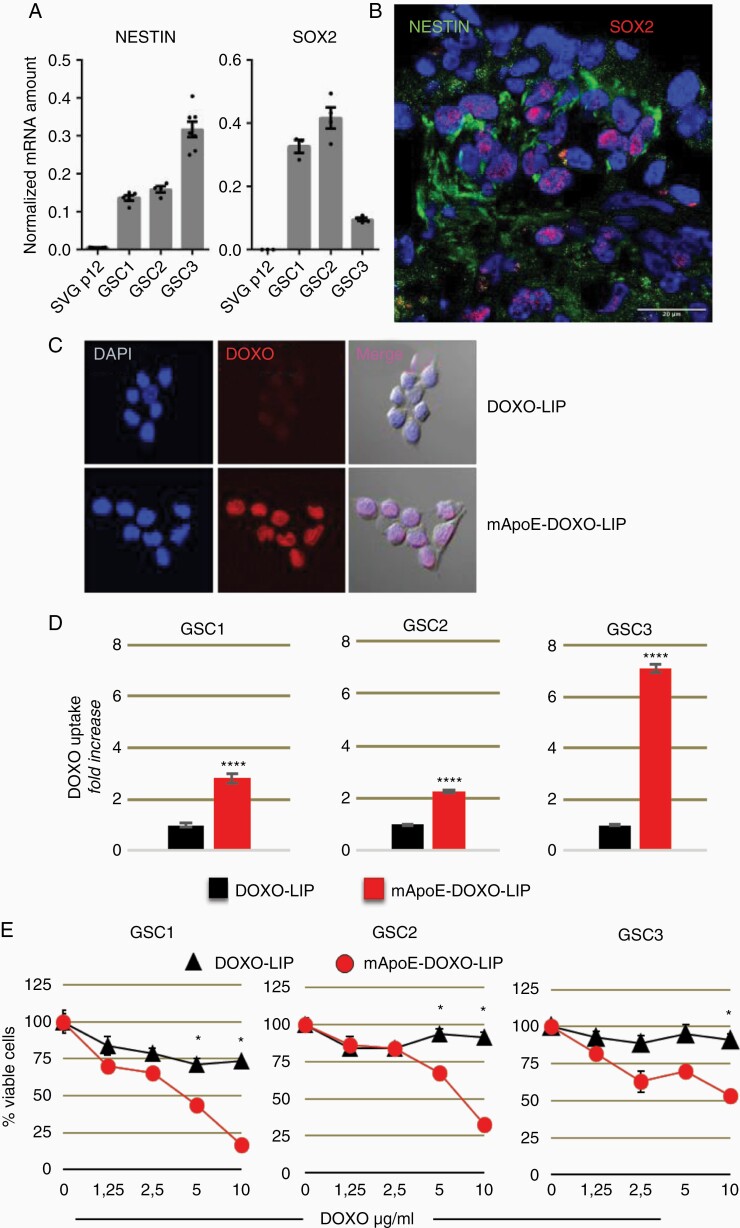
LIP internalization and cytotoxicity in patient-derived GSC lines. Established GSC lines were investigated for stemness: (A) Real-time PCR detection of *NESTIN* and *SOX2* genes in GSC lines and human astrocyte cell line (SVGp12) included as controls. Expression data were normalized on *GAPDH*. Results are shown as mean values ± SEM of triplicates; (B) Representative immunofluorescence of stem markers Nestin (green) and SOX2 (red) in GSC1 xenografts at day 80 from intracranial injection. Nuclei are counterstained with DAPI. Scale bar: 20 μm. (C) Representative images of GCS3 cells incubated with mApoE-DOXO-LIP or DOXO-LIP. (D) Quantification of nuclear DOXO as mean fluorescence intensity (MFI) ± SE normalized to cells incubated with DOXO-LIPs (4 h, DOXO 4 mg/ml). (E) GSC viability after 48 h incubation with LIPs at increasing DOXO concentrations. Values are expressed as mean percentage survival (6 replicates ± SE) normalized to corresponding untreated. *P < .05, ****P < .0001.

To investigate whether mApoE-LIPs could be exploited to target the tumor stem cell subpopulation, 3 GSC lines (IDH1/2 wt) established from patient surgical samples were selected, based on their responsiveness to DOXO: cells with high (GSC1) or moderate (GSC2) sensitivity to DOXO and nonresponder cells (GSC3). Extreme limiting dilution assay (ELDA)^25^ indicated a self-renewal capacity at the following frequencies: 1/3,74 for GSC1, 1/5,21 for GSC2, and 1/4,22 for GSC3 cell lines. Molecular profiling indicated that all the GSC lines did not display a firm pro-neural (PN) or mesenchymal (MES) polarized gene signature. Yet, GSC1 and GSC2 signatures were slightly enriched with PN genes (mesenchymal indexes: −0.39, −0.45 respectively), while the GSC3 culture was characterized by a dim MES signature (mesenchymal indexes: +0.35) (Supplementary Figure S3). The possibility to properly exploit the GSC cell lines was confirmed by the formation of the tumor mass and the preservation of stemness 80 days after their injection into the right striatum of 4-week-old male NOD/SCID mice (Figure 2A and B).

The three patient-derived cell lines were first tested in vitro for their ability to internalize the functionalized LIPs. mApoE-DOXO-LIP intracellular uptake was evaluated through nuclear DOXO fluorescence quantification. DOXO-LIP conjugation with mApoE significantly favored nuclear DOXO accumulation in all three GSC cultures (Figure 2C and D). Dose-dependent cytotoxicity was observed when GSCs were exposed to mApoE-targeted LIPs indicating that embedded DOXO was functional and effective. Conversely, nontargeted DOXO-LIPs did not affect GSC viability, even at the highest DOXO dosage, thus excluding cellular adverse responses to endotoxin contaminants possibly present in the LIP preparations. The highest levels of nuclear DOXO detected in GSC3 cells were not paralleled by a more prominent inhibition of cell viability, consistent with the GSC3 intrinsic drug-resistance to DOXO (Figure 2E).

These data indicate that the GSC lines isolated from patients efficiently internalize mApoE-functionalized LIPs and undergo DOXO-induced cytotoxicity.

### Effects of GSC Irradiation on mApoE-DOXO-LIP Delivery

As radiation therapy (RT) is the gold standard for GBM, we evaluated whether radiation could impact on mApoE-nanovector GSC targeting by modulating LDLR expression.

**Figure 3.**
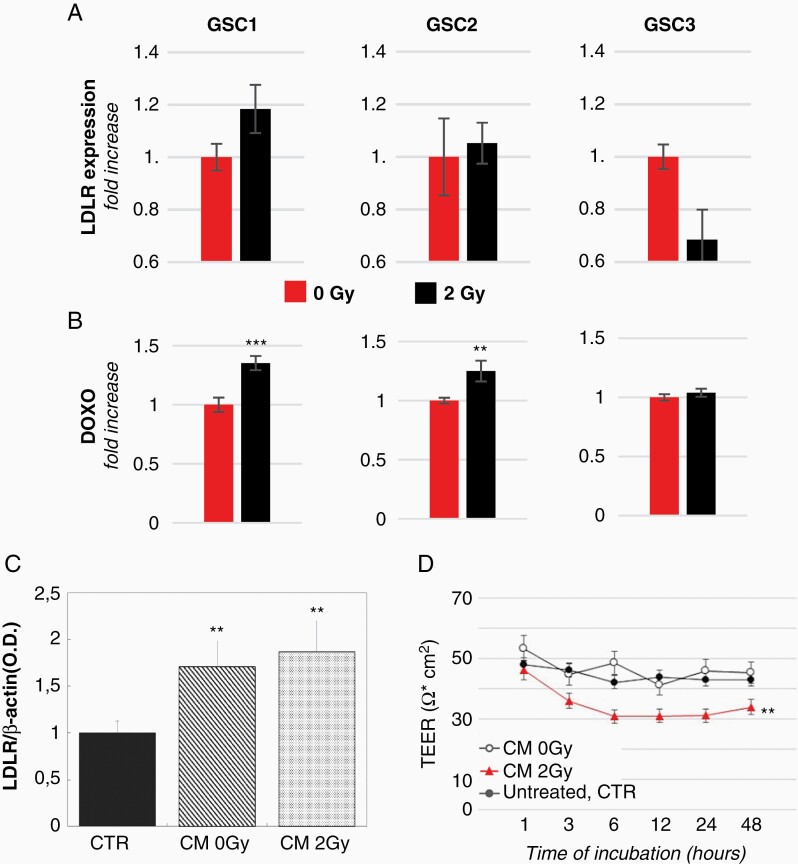
Effect of radiations on LDL-R expression and BBB permeability. (A) Comparison of LDLR protein level in irradiated (2Gy) *vs* nonirradiated (0Gy) GSCs (GSC1: p8; GSC2: p7; GSC3: p9) evaluated by Western-blot. The quantitative analysis was carried out by normalizing LDLR intensity against total proteins. The results are expressed as mean of LDLR optical density fold change of irradiated GSCs *versus* nonirradiated cells from 3 independent experiments ± SE. (B) Intracellular DOXO quantification in nonirradiated and irradiated GSCs incubated with mApoE-DOXO-LIPs. Results are expressed as MFI normalized to MFI of nonirradiated cells ± SE. (C) LDLR protein level in hCMEC/D3 cells before (CTR) and after 12 h-incubation with CM from nonirradiated and irradiated GSCs. (D) TEER values of BBB transwell model untreated (CTR) or incubated with GSC conditioned media (CM) before (0Gy) and after radiation (2Gy). *P < .05, **P < .01, ***P < .001.

Upon 2Gy irradiation, an overall increase of LDLR expression was observed in GSC1 (~18%) and GSC2 (~5%) whereas an inhibition of expression was observed in GSC3 cell line (Figure 3A). The radiation-induced LDLR expression was paralleled by higher levels of mApoE-DOXO-LIP uptake by irradiated GSC1 and GSC2 cells but not GSC3 (Figure 3B). Interestingly, immunohistochemistry analyses performed on a cohort of single cells confirmed the extent of LDLR induction in GSC1 cells. Conversely, a much higher LDLR induction in GSC2 compared to GSC1 cells, and no alteration in GSC3 cell was observed suggesting a heterogeneous response to radiation among GSCs (Supplementary Figure S4).

We next investigated whether irradiated tumor cells could alter BBB properties. Incubation of the transwell BBB model with the GSC conditioned media (CM) from nonirradiated (0Gy) and irradiated (2Gy) cells induced an upregulation of LDLR expression on endothelial hCMEC/D3 cells (Figure 3C). Though, only CM from irradiated GSCs caused a stable drop of TEER indicating an increased BBB permeability (Figure 3D).

### mApoE-DOXO-LIP Antitumor Activity in Orthotopic GSC-PDXs

**Figure 4.**
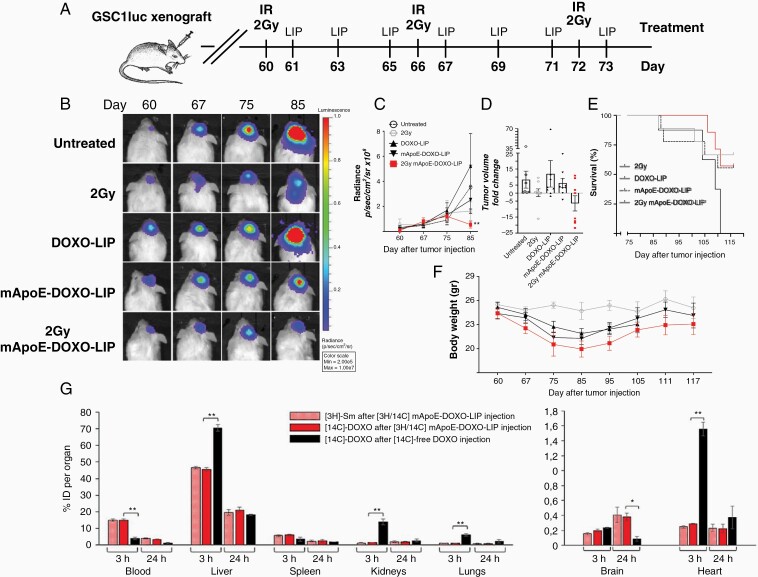
In vivo efficacy of mApoE-DOXO-LIPs in the GSC-PDX model. (A) Treatments started 60 days (D60) after intracranial injection of GSC1luc cells and were administered according to the indicated schedule. GSC1luc bearing mice (*n* = 9 mice/group) received 35 μg/dose of encapsulated DOXO intraperitoneally. Irradiation (2Gy) was administered as whole-brain treatment. (B) Representative BLI imagines of tumor-bearing mice untreated (CTR), and treated with untargeted DOXO-LIPs (DOXO-LIP), mApoE-functionalized LIPs as single agent (mApoE-DOXO-LIP) or concomitant with radiation (2Gy/mApoE-DOXO-LIP). (C) Time course quantification of tumor BLI signals. Results are expressed as mean of radiance values ± SE detected at the indicated time points. *P*-values are calculated using two-way ANOVA and Tukey's multiple comparisons test (**P <.01). (D) Tumor growth/inhibition at D85 (12 days after treatment end). Data are expressed as relative BLI radiance mean values ± SE normalized to BLI values at D75. (E) Kaplan–Meier survival curve. *P*-log = 0.0863. (F) Body weights of the alive animals at the indicated time points. Data are presented as the mean values ± SE. (G) mApoE-DOXO-LIP distribution in peripheral organs and across intact BBB. Dually radiolabeled mApoE-DOXO-LIP ([^3^H]-Sm/[^14^C]-DOXO) or free-[^14^C]-DOXO were intravenously injected in healthy mice. Mice were sacrificed 3 h or 24 h after the injection. Radioactivity in peripheral organs was measured by liquid scintillation counting. The amount of [^3^H]-Sm or [^14^C]-DOXO is expressed as percentage of injected dose (ID) ± SD, *P <.05, **P < .01.

The in vitro results strongly suggested that RT may facilitate LIP brain delivery and uptake by tumoral cells. Therefore, mApoE-DOXO-LIPs were delivered either as single treatment or in combination with radiations to GSC1-PDX obtained by orthotopic injection of luciferase-transfected GSC1 (GSC1luc) in NOD/SCID mice. GSC1 were chosen based on higher sensitivity to DOXO compared to GSC2, both as free drug (in vitro IC_50_: 0,4 *vs* 1 μg/ml, Supplementary Figure S3) and encapsulated into LIPs (in vitro IC_50_: <5 *vs* >5 μg/ml, Figure 2E). Eight weeks after intracranial injection (D60), mice were homogenously grouped (9 mice/group) based on bioluminescence imaging (BLI). mApoE-functionalized LIPs as single agent (mApoE-DOXO-LIP) or in combination with radiation (2Gy/mApoE-DOXO-LIP) were administered according to the schedule reported in Figure 4A. Untreated mice (CTR), and mice treated with untargeted LIPs (DOXO-LIP) or radiation only (2Gy) were included as controls. All mice received a cumulative dose of liposomal DOXO below the maximal tolerated dose (15–20 mg/kg)^26^ of 10.5 mg/kg (245 μg/mouse subdivided in 7 doses) corresponding to a dosage of 35 mg/m^2^, approximately half of the 60 mg/m^2^ dose commonly used in humans.

BLI performed during (D67) and at the end (D75) of the treatment (Figure 4B) indicated an overall low tumor growth rate that underwent a considerable boost in the subsequent 10 days (D85) reaching an 8.4 ± 5.3 and 12.0 ± 8.2 fold increase, in untreated and DOXO-LIP controls, respectively. Treatment with mApoE-DOXO-LIP slowed tumor expansion to a 6.3 ± 2.7 fold increase (Figure 4C and D). Importantly, the highest and significant level of tumor growth inhibition, −6.4 ± 4.7 times, was observed in mice receiving radiation together with mApoE-DOXO-LIPs (2Gy/mApoE-DOXO-LIP) (Figure 4B–D). Accordingly, overall survival equaled tumor expansion/inhibition. Despite the lack of statistical significance, higher survival was observed upon the 2Gy/mApoE-DOXO-LIP treatment compared to any other treatments (Figure 4E).

Body weight monitoring indicated a loss of weight, approximately 12%, in all mice receiving LIPs. Weight loss stopped in concomitance with the end of the treatment (D75) and since then mice started gaining weight regardless of tumor volume and death event (Figure 4F) suggesting a sensible, yet compatible with life, LIP systemic toxicity. To gain more insights into LIP toxicity, mApoE-DOXO-LIP biodistribution was investigated in healthy mice. Dually radiolabeled mApoE-DOXO-LIPs or radiolabeled free-DOXO were intravenously administered. Three and 24 h after injection, radioactivity was measured. Results (Figure 4G) showed that DOXO incorporation into mApoE-LIPs increases its circulation half-life, as indicated by the higher radioactivity levels in the peripheral blood after injection of mApoE-[^14^C]-DOXO-[^3^H]-SmLIPs compared to free-[^14^C]-DOXO. Moreover, [^14^C]-DOXO encapsulation significantly reduced its accumulation in liver, kidneys, lungs, and heart. Nonetheless, the liver remains the main organ where approximately 40–50% of the injected LIPs accumulate. Consistent with the observation that nanoparticles accumulate in the brain over time,^27^ a progressive increase of radioactivity was detected upon mApoE-DOXO-LIP delivery. Results indicated that 24 h after injection the radioactivity of free-[^14^C]-DOXO in the brain was reduced by 2.7 folds whereas it was 4.4-fold higher after the administration of mApoE-[^14^C]-DOXO-[^3^H]-LIP. Yet, low levels of radioactivity were detected in the brain suggesting a limited mApoE-DOXO-LIP crossing of the intact BBB.

### mApoE-DOXO-LIPs Elicit Tumor Cell Death by Apoptosis

To investigate the cause of tumor growth inhibition in mApoE-DOXO-LIP-treated mice, BLI experiments were corroborated by histological analyses. To this end, GSC1-luc were injected in an additional group of mice, that were treated as before (Figure 4A). Likewise previously, tumor growth was monitored by BLI and a comparable tumor growth rate was observed between the 2 experiments (Supplementary Figure 5). Mice were sacrificed at the end of the treatments (D75).

**Figure 5.**
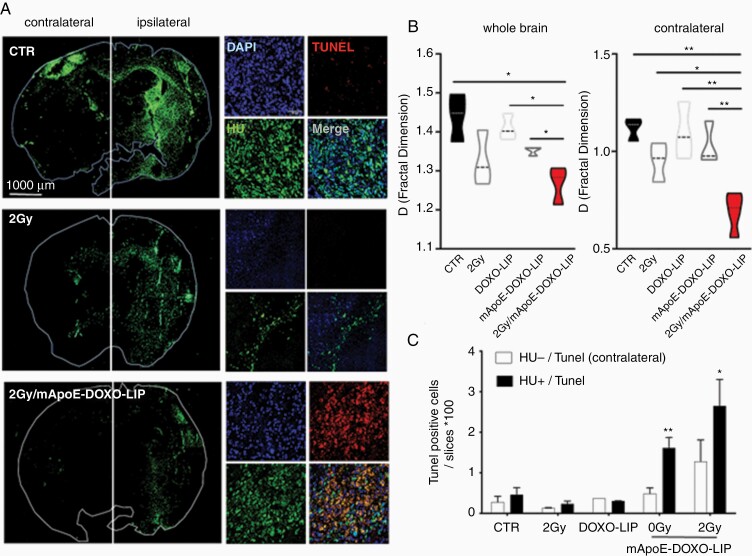
Tumor growth and apoptosis in mApoE-DOXO-LIP treated GSC-PDX. Mice (*n* = 4 mice/group) treated as previously described (Figure 4A) were sacrificed at D75 and brains collected for histological analyses. (A) Representative coronal sections from untreated (CTR), irradiated (2Gy), and 2Gy/mApoE-DOXO-LIP treated mice. GSC1 cells were detected by anti-human nuclei (HU) immunofluorescence. Apoptotic cells were detected by TUNEL staining on the same brain sections. (B) Quantification of GSC1 cells detected as HU-positive cells. Imaging analysis was carried out using the FracLac plugin of the FIJI software. Three serial sections for each brain were analyzed. Results are expressed as mean of fractal indexes ± SE. One-way ANOVA: whole brain: *P* = .0122; contralateral: *P* = .0013. Comparison between 2 groups was performed by Student's t-test. (D) Quantification of apoptotic cells defined by nuclear HU, DAPI, and TUNEL colocalization. Results are expressed as mean values ± SE of HU-positive (human, black bars) and HU-negative (murine, white bars) cells. Only murine cells in the contralateral hemisphere were considered. Imaging analysis was carried out as in panel B. Two-way ANOVA and Bonferroni *post hoc* testing were used to assess differences among treatments (**P < .01). Comparison between the 2Gy group and the mApoE-DOXO-LIP groups, with and without radiation, was performed by Student's t-test. *P < .05, **P < .01.

Xenograft histology confirmed tumor shrinkage in mice receiving radiations and mApoE-DOXO-LIPs (Figure 5A and Supplementary Figure S6). Overall, GSC1 tumors treated with mApoE-DOXO-LIP and radiation (2Gy/mApoE-DOXO-LIP) displayed the lowest level of brain infiltration. Interestingly, GSC1 cell quantification in the contralateral, not-injected hemisphere revealed a significant reduction of tumor cell spreading along commissural fibers upon 2Gy/mApoE-DOXO-LIP treatment (Figure 5B) indicating a significant lower tumor invasion and, thus progression.^28^

Apoptosis analysis by in situ terminal deoxynucleotidyl transferase dUTP nick end labeling (TUNEL) assay indicated a colocalization between TUNEL and HU staining (Figure 5A). Significantly, apoptotic GSC1 cells were observed exclusively in the xenografts of mice receiving mApoE-DOXO-LIPs, both as single agent (0Gy/mApoE-DOXO-LIP) or concomitant with radiation (2Gy/mApoE-DOXO-LIP). No/low TUNEL staining was observed in mice treated with radiation alone (2Gy) and in HU-negative nuclei (murine cells) (Figure 5C).

Besides the brain, peripheral organs were collected from all the mice at the moment of the sacrifice. Consistently with the biodistribution data (Figure 4G) mice treated with mApoE-DOXO-LIPs displayed signs of liver distress (steatosis) whereas hearts, spleens, and kidneys appeared histologically normal (Supplementary Figure S8).

### Immune Cell Death and GAMM Phagocytosis of Apoptotic GSCs

In line with the knowledge that DOXO can trigger ICD, the immunogenicity of apoptotic cells was evaluated by means of eukaryotic initiation factor 2α (eIF2α) phosphorylation, a hallmark of ICD.^29^ The quantification of phospho-eIF2α showed a significant higher number of positive tumoral cells in 2Gy/mApoE-DOXO-LIP compared to untreated mice.

**Figure 6.**
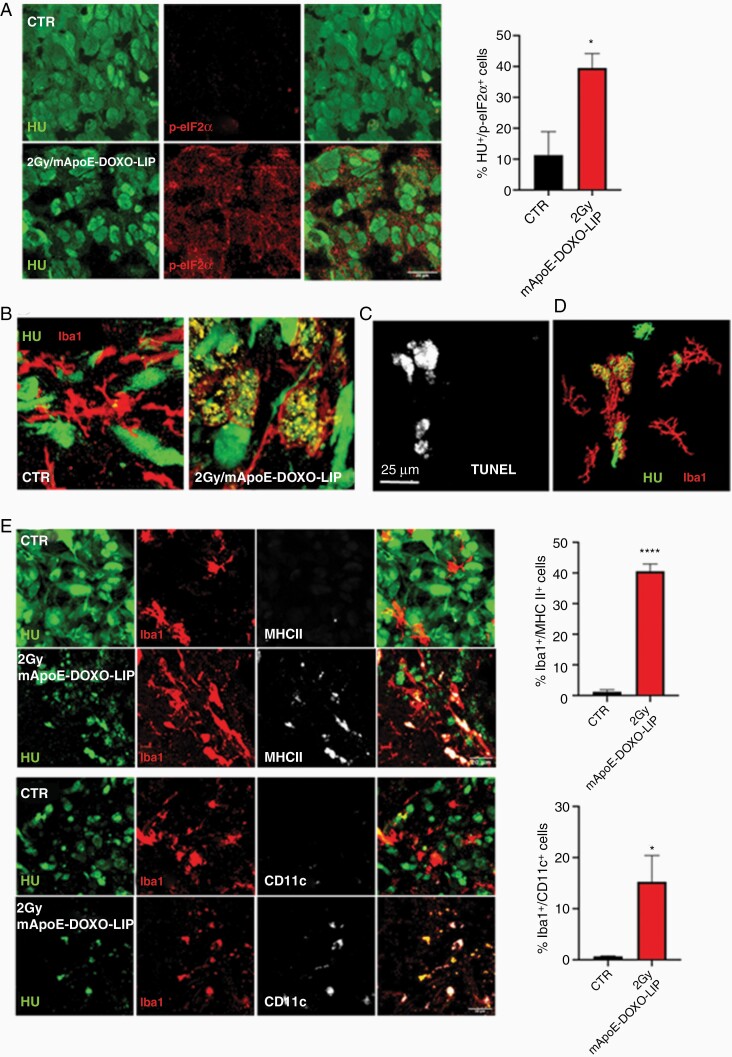
GSC cell death and immune activation. (A) Detection and quantification of the ICD marker phospho-eIF2α (p-eIF2α) in GSC1tumors, Human Nuclei (HU) positive cells, from untreated (CTR) and 2Gy/mApoE-DOXO-LIP treated mice. p-eIF2α quantification was carried out on 3 brain coronal images each condition acquired using a DMi8 fluorescent microscope and a Leica Application Suite X (LAS X) imaging system (Leica Microsystems). (B) Representative confocal images of Iba-positive, tumor infiltrating microglia/macrophage in untreated (CTR) and 2Gy/mApoE-DOXO-LIP treated mice. (C and D) *In situ* TUNEL detection within Iba1-positive cells in GSC tumor border and 3D rendering of panel C; (E) Representative confocal images and quantification of the dendritic markers MHCII and CD11c in Iba1-positive cells in untreated (CTR) and 2Gy/mApoE-DOXO-LIP treated mice. Quantification has been performed as in panel A. *P < .05, ****P < .0001.

Immunogenic dead cells expose on the surface and release different molecules to attract and interact with the cells of the innate immunity, macrophage, and dendritic cells primarily.^18^ As glioma-associated microglia/macrophages (GAMMs) heavily infiltrate GBM,^30^ their recruitment at the tumor site was evaluated by immunofluorescence using the myeloid marker Iba1. Results showed no relevant difference in the extent of GAMM infiltration among treatments (Supplementary Figure 7A). Conversely, a conspicuous difference in the morphology of Iba1-positive cells was observed. In untreated tumors, Iba1-positive cells displayed a ramified, highly branched shape disclosing a resting status. In contrast, in mApoE-DOXO-LIP tumors Iba1-cells had an amoeboid morphology. Moreover, HU-positive fragments, consistent with GSC1 debris released by apoptotic cells, were detected inside Iba1-positive cells of mApoE-DOXO-LIP-treated mice indicating a phagocytic activity (Figure 6B; Supplementary Figure 7B). Of note, GAMM amoeboid morphology and HU-positive fragments colocalized with TUNEL staining. As a further support, Iba1-positive cells associated to vital TUNEL-negative GSC1 cells had a branched morphology (Figure 6C and D).

It is known that the engulfment of dead cells triggers macrophage conversion into professional antigen presenting cells.^18^ Therefore we investigated the expression of the dendritic markers MHCII and CD11c^31^ in the Iba1-positive cells. No MHCII and CD11c induction was detected in resting microglia/macrophages from untreated mice, whereas MHCII and CD11c were significantly upregulated in activated GAMM upon mApoE-DOXO-LIP/radiation combined treatment (Figure 6E) supporting tumor antigen cross presentation.

## Discussion

Current standard of care is not successful in delivering effective and persistent treatments to GBM. Several elements concur to therapy failure, including the high amount of chemo-, radio-resistant tumor-initiating GSCs, and the presence of intact BBB in the peripheral niches.^32^ To approach these issues, we investigated LIPs functionalized with mApoE peptide, known to enhance BBB crossing by transcytosis,^33^ as drug nanovehicles to deliver DOXO into the brain and to target GSCs.

LIP preparations, including the ones used in this study, are stable at slightly acidic pH (6.5), the typical value of tumor environment, inferring the necessity of a targeting ligand on LIP surface to vehicle and release the embedded payload inside GBM cells. We demonstrated that after in vitro BBB crossing, mApoE-DOXO-LIPs remain intact and able to impact the viability of GBM cells. DOXO nuclear accumulation occurred only in the presence of mApoE-functionalized LIPs and was significantly inhibited by the incubation with endocytosis inhibitors, thus indicating a targeted delivery rather than a passive intracellular diffusion of DOXO. mApoE-specific, receptor-mediated uptake, and cytotoxicity was confirmed in patient-derived GSCs, pointing out the fundamental role of mApoE in conferring efficacy to untargeted LIPs and strongly supporting the indication of mApoE as a valuable targeting moiety for GSCs.

In irradiated (2Gy) GSCs the higher level of LDLR expression was associated with an increased cellular internalization of mApoE/DOXO/LIPs. Moreover, we observed that irradiated GSCs have the capacity to modify neighboring BBB in terms of permeability and induction of LDLR expression on endothelial cells that could favor transcytosis. As in vivo biodistribution indicated that healthy BBB allowed a modest accumulation of mApoE-DOXO-LIPs into the brain questioning a possible therapeutic efficacy, radiations could be instrumental to heighten nanovector brain delivery and GSC uptake at a therapeutic level. Moreover, a low crossing rate through intact BBB combined to a higher delivery rate in the irradiated field, would confer tumor selectivity to mApoE-conjugated and drug-loaded nanovectors protecting healthy brain parenchyma from off-target effects. This is relevant considering that GBM patients receive RT limited to the surgical margins where most of the recurrences originate due to the presence of infiltrating GSCs.^34^

mApoE-DOXO-LIP anti-tumor activity was evaluated in GSC-PDXs obtained by intracranial injection of patient-derived GSCs in NOD/SCID mice, the in vivo experimental model that ensures stemness maintenance.^35^ This model generated slow-growing, diffused tumors with elevated invasion capacity, proved by the migration of the injected GSC1luc cells into the nontransplanted hemisphere through commissural fibers. In this context, untargeted DOXO-LIPs did not affect tumor growth. Most importantly, mApoE-targeted, but not untargeted, DOXO-LIPs triggered a significant level of apoptosis in GSC xenografts. Nevertheless, the most significant tumor inhibition associated with increased survival and highest level of apoptosis was achieved only by the concomitant radiation and mApoE-DOXO-LIP treatment (2Gy/mApoE-DOXO-LIP).

It is conceivable that despite mApoE-DOXO-LIPs cross the BBB and target GSCs, yet their accumulation and diffusion into the brain parenchyma did not reach an adequate tissue concentration able to cause steady effects. Radiation, other than inducing LDLR expression and BBB permeability alteration,^3,36,37^ it is known to activate matrix metalloproteinases^38^ causing extracellular matrix degradation that would facilitate LIP diffusion in the tumor microenvironment after extravasation.

Interestingly, a noticeable tumor inhibition was achieved by the radiation treatment (2Gy). Crucially, however, radiation alone did not trigger apoptosis. Therefore, tumor inhibition by radiation has to be ascribed to cytostatic, rather than cytotoxic, effects affecting GSC proliferation but not viability.

DOXO-loaded LIPs caused a slight systemic toxicity, most likely due to an excessive liver accumulation. However, LIP toxicity was reverted by the end of the treatments and, despite the frailty of NOD/SCID mice, did not cause life threatening conditions. Noteworthy, encapsulation significantly reduced DOXO accumulation in the principal peripheral organs (liver, kidney, lung, heart), while increased the level of circulating DOXO and brain accumulation.

Moreover, mApoE-DOXO-LIPs did not affect GAMM viability and phagocytic activity, demonstrating the mApoE specific uptake by GSCs and the absence of brain off-target effects. Additional pharmacokinetic and pharmacodynamic investigations are needed to establish a more appropriate treatment protocol to balance therapeutic and side effects.

In conclusion, here we are proposing the adjuvant use of mApoE-functionalized LIPs combined to RT as a promising approach to overcome BBB impediments and deliver DOXO to GSCs. Likewise DOXO, other drugs and/or molecules with proved anti-GBM activity, and particularly those excepted by BBB efflux pumps, could be encapsulated into mApoE-LIPs to boost their brain delivery. The encapsulation of different and/or multiple drugs would be beneficial to contrast GBM heterogeneity. Indeed, we reported difference among GSC lines in the response to DOXO.

Yet, the use of DOXO has valuable advantages to consider. DOXO is one of the most commonly used chemotherapeutic agent. It is known to display excellent antineoplastic activity in GBM cells in vitro but poor efficacy in vivo due to extrusion by multidrug resistance-related proteins present on both BBB and GBM cells.^39^ To constrain DOXO severe adverse effects such as cardiotoxicity and myelosuppression FDA-approved liposomal DOXO formulations are currently in use in the clinic. However, the lack of surface functionalization^40^ precludes their use for GBM treatment, due to their absent/inefficient BBB crossing. We demonstrated that the simple conjugation of the mApoE peptide on liposomal DOXO formulations could efficiently overcome the impediments handed by the BBB, including drug resistance by molecular extrusion. Moreover, radiation proved a key factor in order to achieve mApoE-DOXO-LIP therapeutic accumulation and diffusion within the GBM tumor area.

It is relevant to highlight the low level of MGMT promoter methylation (2%) in GSC1 cells advocating mApoE-DOXO-LIPs as an alternative therapeutic option to circumvent temozolomide resistance.

Last but not least, DOXO is one of the few chemotherapeutics that triggers immunogenic apoptosis in tumor cells^41^ and our results indicate that this is true also for GBM. This is of fundamental importance for in situ shaping and restoring anti-tumor immune response particularly when immunotherapy approaches are pursued.^42^

## Supplementary Material

vdab076_suppl_Supplementary_Materials

## Notes

**Authorship Statement**. M.P., F.R., M.Mat., L.P. conceived the experimental design, analyzed the data, prepared the manuscript. All authors reviewed the manuscript. F.R., B.F., M.Mas. synthetized and characterized drug-loaded liposomes. M.F. contributed with TEM analysis. B.F., E.L., M.G., F.R. performed the experiments with the in vitro BBB model. M.P., A.P., F.DiM. established patient-derived GSC primary lines. M.T., F.U. generated luciferase stable transfected GSC cell lines. M.P., M.T. generated orthotopic xenografts and treated them. M.P. conducted BLI and histological tumor imaging. M.P., E.S., S.R. performed cell culture, MTT assay, immunohistochemistry and imaging analyses.
