# Supplementary material for: Radiation and adjuvant drug-loaded liposomes target glioblastoma stem cells and trigger in-situ immune response
Source: Neurooncol Adv. 2021 Jun 18;3(1):vdab076. doi: 10.1093/noajnl/vdab076 (PMC8349181; doi:10.1093/noajnl/vdab076)
Supplement: vdab076_suppl_Supplementary_Materials [file vdab076_suppl_supplementary_materials.pdf]

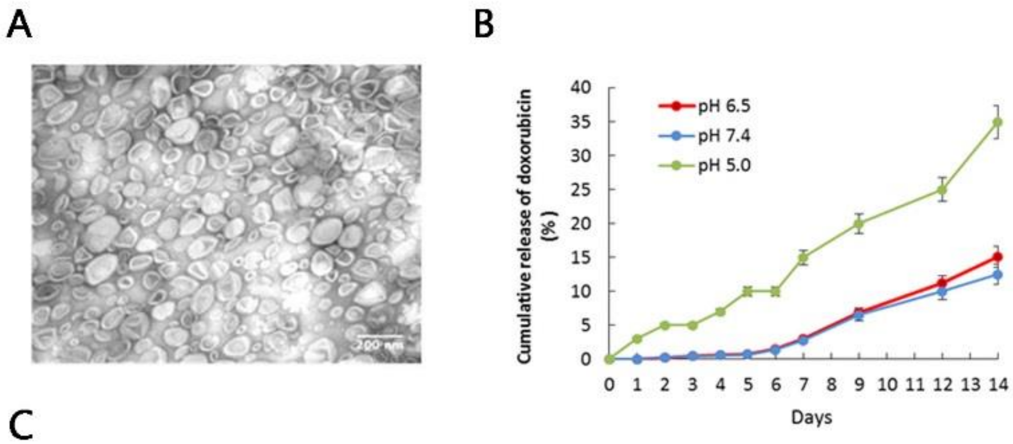

C

Sample	Size ± SD (nm)	Polydispersion Index (PDI)	ζ-pot ± SD (mV)
**LIP**	102 ± 5	0.077	-25.51 ± 0.82
**DOXO-LIP**	117 ± 12	0.106	-18.74 ± 1.25
**mApoE-DOXO-LIP**	128 ± 15	0.081	-12.48 ± 2.02

Supplementary Figure S1. Characterization of mApoE-DOXO-LIPs.

(A) Representative TEM image of mApoE-DOXO-LIP. Scale bar: 200 nm. Transmission electron microscopy analyses demonstrated that mApoE-DOXO-LIPs are predominantly unilamellar structures with a mean diameter of 113.33 ± 22.88 nm (B) Cumulative DOXO release (percentage) over time from mApoE-DOXO-LIPs at pH 5.0, 6.5 or pH 7.4, at 37 °C. The reported data are the mean of at least five different measurements ± SD. (D) Physicochemical parameters of synthetized LIPs: mean diameter (size), polydispersion index (PDI) by dynamic light scattering (DLS); Zeta-potential (ζ-pot) by Zeta-potential analyzer. . All liposome preparations maintained a constant size and ζ-potential for up to 10 days within the experimental error (<2.6% of variation). The yield of DOXO encapsulation into LIP was 95 ± 3% and the final preparation contained 220-± 12 µg of DOXO/µmol of lipids. The yield of LIP surface functionalization with mApoE peptide ranged between 55% and 65% corresponding to 1.25% of total lipids

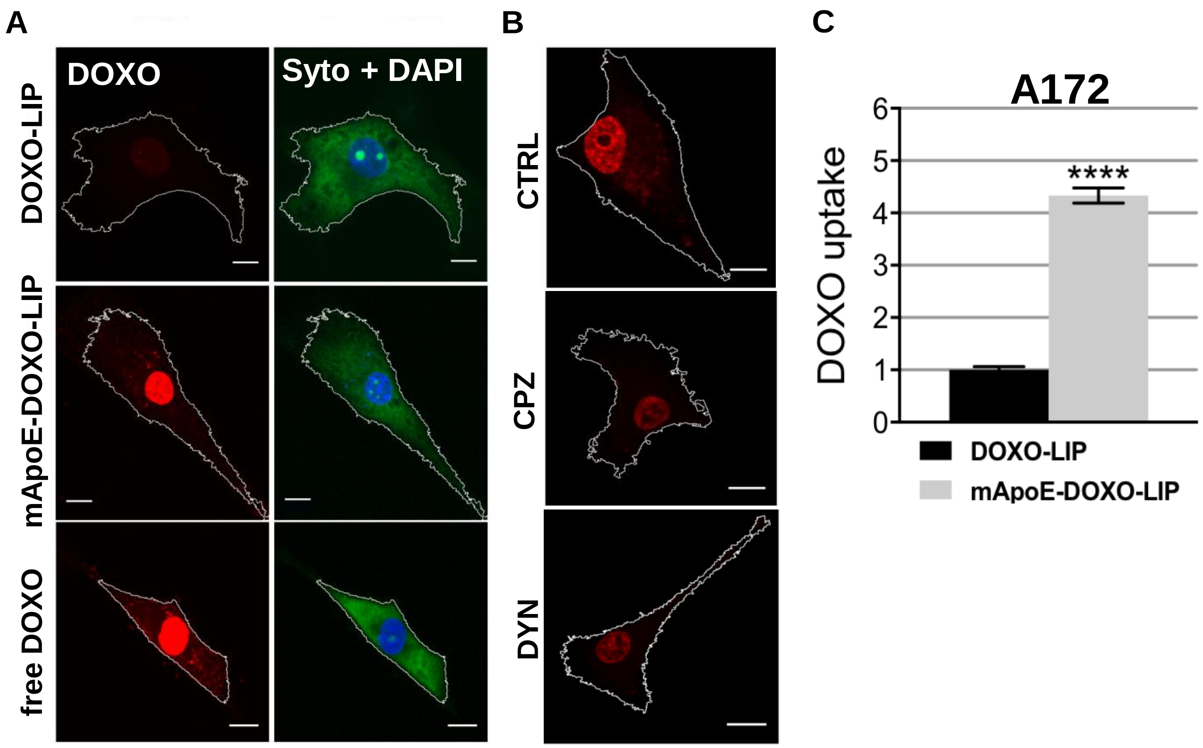

Supplementary Figure S2. LIP internalization in GBM cells.

(A) Maximum projections of DOXO and Syto/DAPI merged signals acquired by means of confocal microscopy in U87MG cells incubated with liposomes (DOXO-LIP or mApoE-DOXO-LIP) or free-DOXO. (B) Maximum projections of DOXO signals in U87MG cells incubated with mApoE-DOXO-LIP alone (CTRL) or in the presence of the endocytosis inhibitors chlorpromazine (CPZ) and dynasore (DYN). (D) Nuclear DOXO quantification in the sample incubated with mApoE-DOXO-LIP in the presence/absence of endocytosis inhibitors. Scale bar 10 µm. (C) Nuclear fluorescence quantification (DOXO uptake) in A172 cells incubated with the indicated liposomes. refers to the mean fluorescence values were normalized by the sample incubated with DOXO-LIPs. **** P<0.0001.

GBM cells (U87-MG, A172) were seeded on glass coverslips at a concentration of 3500 cell/cm2. The day after the cells were incubated with liposomes (DOXO-LIP or mApoE-DOXO-LIP) or free-DOXO (DOXO, 4 µg/ml in every condition) for 4 h in complete medium with 5% FBS. In the experiments with the endocytosis inhibitors, CPZ (10 µM) or DYN (80 µM) were added to the cell medium one hour before the incubation with liposomes. The inhibitors were kept into the cell medium during the whole experiment (5-hour incubation). During the last 45 min of liposomes incubation (with or without endocytosis inhibitors) the cells were loaded with Syto45 (Molecular Probe) then washed with PBS, fixed in PFA 4% in PBS for 15 min, DAPI (Sigma-Aldrich) stained (10 µg/ml in PBS, 30 min) and mounted in 90% glycerol in PBS. The Z-stack series of the cells in different experimental conditions were acquired by means of a confocal microscope (TCS SP5, Leica Microsystems GmbH) with the HCX PL APO 63x/1.4 OIL objective. The DAPI (ex/em: 405/415-450 nm), Syto45 (ex/em: 458/470-500 nm) and DOXO autofluorescence (488/570-700) signals were acquired sequentially at high scan rate. Images were processed using ImageJ (Wayne Rasband, National Institute of Mental Health, Bethesda, Maryland, USA) using the DAPI signal to segment the nucleus and the Syto45 signal to identify the cell contour. The DOXO fluorescence was quantified in the nuclei as mean grey values in the optical section were the nucleus area were maximized.

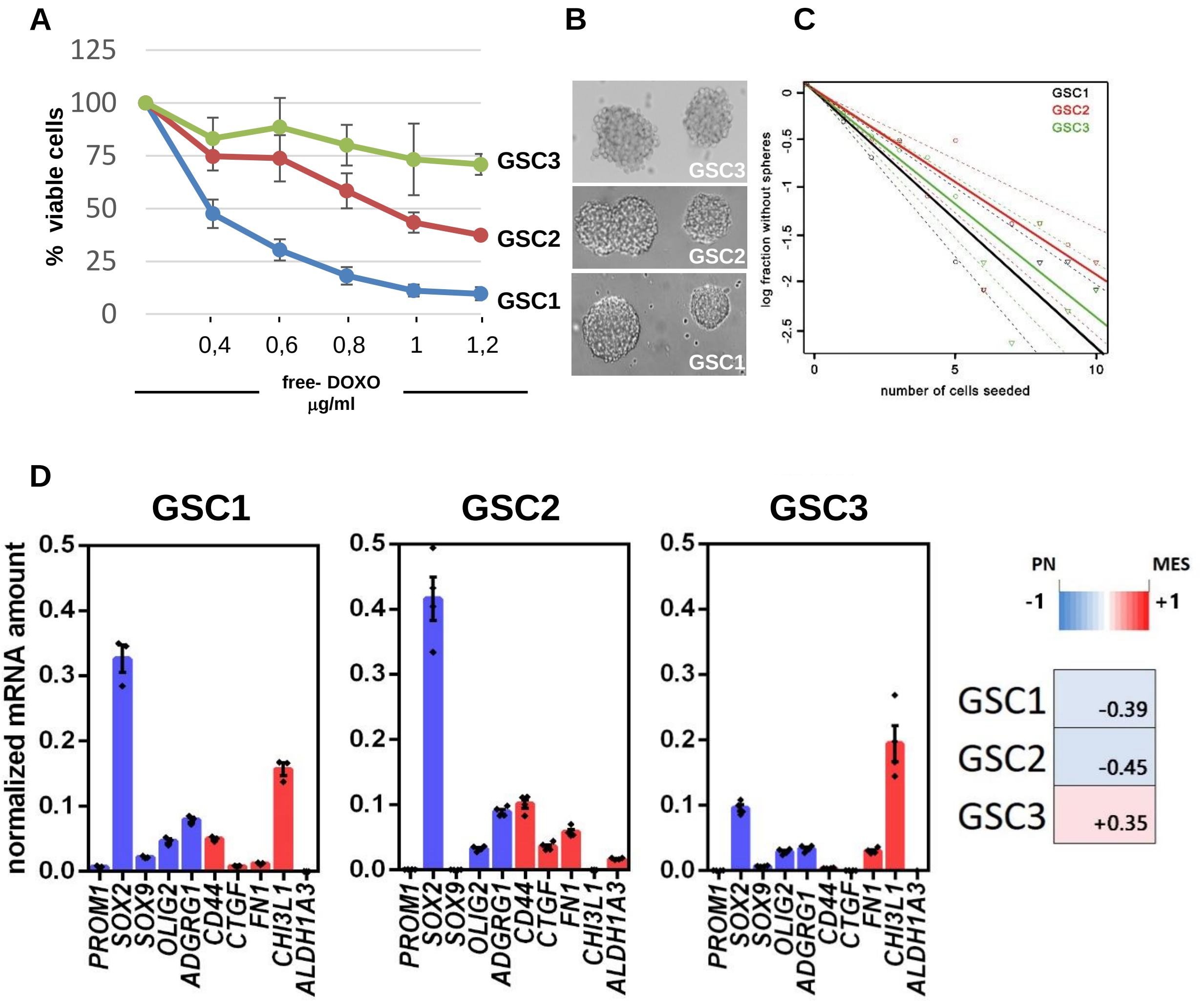

E

% MGMT promoter methylation											
	CpG Island										sample average
	#1	*#2*	*#3*	*#4*	*#5*	*#6*	*#7*	*#8*	*#9*	*#10*	
**GCS1**	1	2	2	2	2	2	3	2	2	2	***2***
**GCS2**	9	11	13	11	9	91	78	70	100	89	***48***
**GCS3**	1	2	1	1	1	2	1	2	2	2	***2***

Supplementary Figure S3. GSC line characterization.

(A) Effect of free-DOXO on GSC cultures viability. Upon treatment with the indicated amount of free-DOXO, cell survival was measured by MTT assay. Cells were exposed to increasing concentrations of free-DOXO for 48 h. The obtained relative values were normalized to the values from the corresponding untreated cells and are shown as percentage survival. Results are expressed as mean percentage of 4 independent experiments performed in triplicate ± SE. (B) Representative bright field images of GSC neurospheres (GSC1and GSC2: culture passage (p) 6; GSC3: p9). (C) *In-vitro* extreme limiting dilution assay (ELDA) testing stem-cell frequency within GSC cultures (p9). ELDA revealed high frequency of stem-cells in all GSC cultures tested and no significant differences among them. (D) Pro-neural (PN) *vs* mesenchymal (MES) molecular subtyping (GSC1and GSC2:p8; GSC3:p6). The PN/MES metagene calculation was performed as previously described (*Bhat KPL et al. Mesenchymal Differentiation Mediated by NF-κB Promotes Radiation Resistance in Glioblastoma" Cancer Cell 2013; 24(3):331-46*). Briefly, metagene score was calculated for each sample and compared to others after Z-score correction, being +1.00 value indicating a complete MES culture and -1.00 a pure PN subtype. Given the score obtained, analyzed cultures don’t displayed a neat and distinguished signature with GSC1 and GSC2 being slightly PN and GSC3 slightly MES. (E) Pyrosequencing analysis of MGMT promoter methylation level. DNA was extracted from cell lines using QIAamp® DNA Mini Kit (QIAGEN, Hilden, Germany). Methilation status has been evaluated by means of MGMT Plus® kit (Diatech Pharmacolgenetics, Jesi (AN), Italy) according to manufacturer’s instructions.

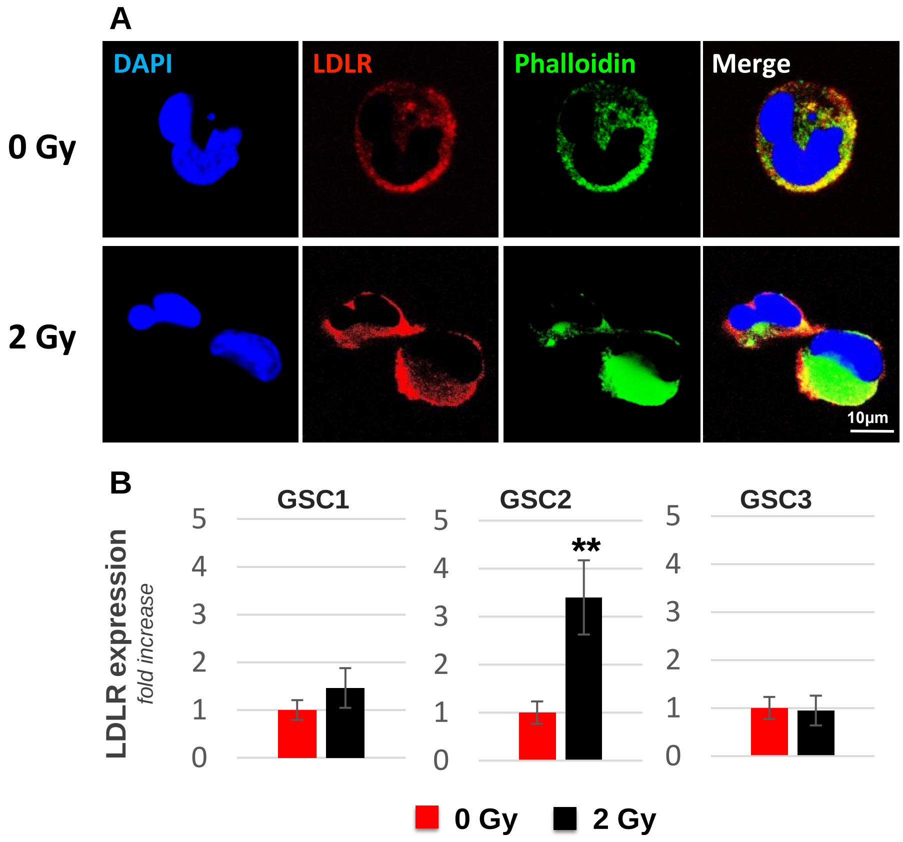

Supplementary Figure S4. LDLR expression on GSCs before and after radiation.

(A) Representative confocal images of irradiated (2Gy) and non-irradiated (0Gy) GSC2 cells (culture passage 7) immunostained for human LDL-R (red). In blue is the nuclear staining (DAPI). The cell borders were defined by Phalloidin staining (green). (B) Quantification of LDLR level of expression in GSCs 24h after irradiation (2Gy). At least fifty cells/sample wereanalyzed. The results are expressed as mean LDLR fluorescent area respect to non-irradiated (0Gy) samples ± SE. Comparison between groups was performed by Student's t-test. **P< 0.01

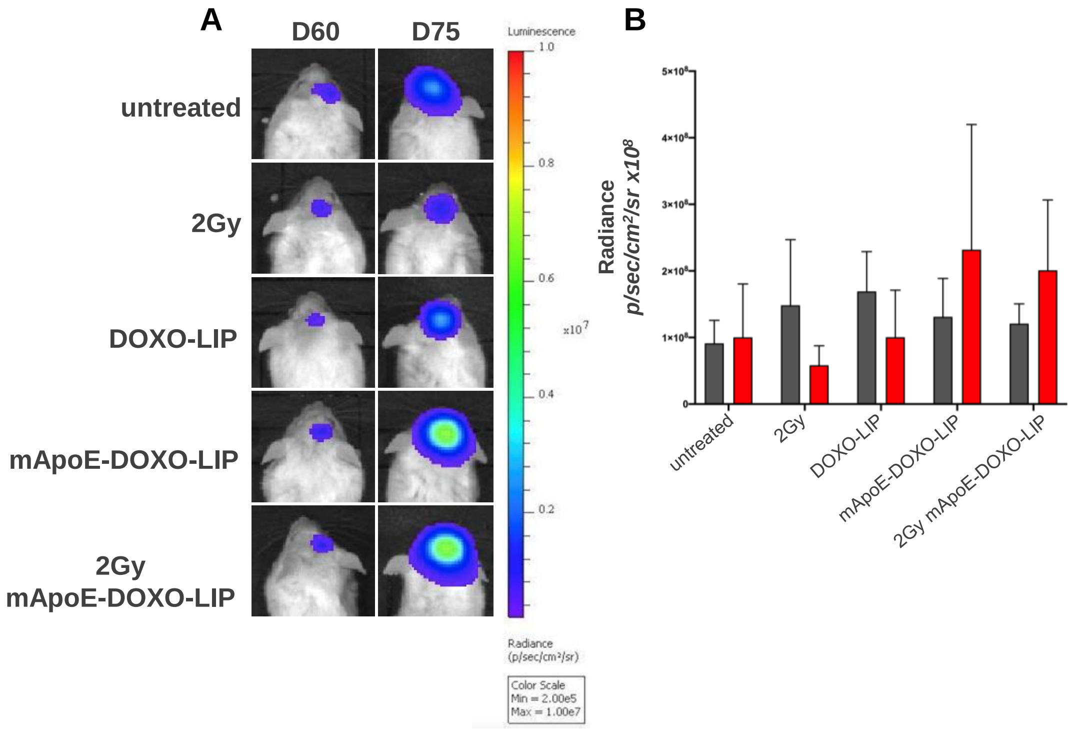

Supplementary Figure S5. BLI monitoring of GSC1luc xenograft growth

GSC1luc cells were injected into the right striatum of NOD/SCID mice. Treatments started 8 weeks after tumor injection (D60) and were carried out as described in Fig. 4. Tumor growth was evaluated by bioluminescence (BLI). At the end of treatments (D75) animals were euthanized with intracardiac perfusion of saline solution, followed by 4% paraformaldehyde. (A) Representative BLI imagines of mice untreated and treated with radiation (2Gy), DOXO-LIPs (DOXO-LIP), mApoE-DOXO-LIPs as single agent (mApoE-DOXO-LIP) or concomitant with radiation (2Gy/mApoE-DOXO-LIP). (B) Radiance value at D75 detected in the groups of mice reported in Fig.4 (gray bars) compared to the values of the second groups of mice utilized for histological analyses and here reported (red bars). Data are roprted as mean values ± SE. No statistical significance was observed among treatment and experiments.

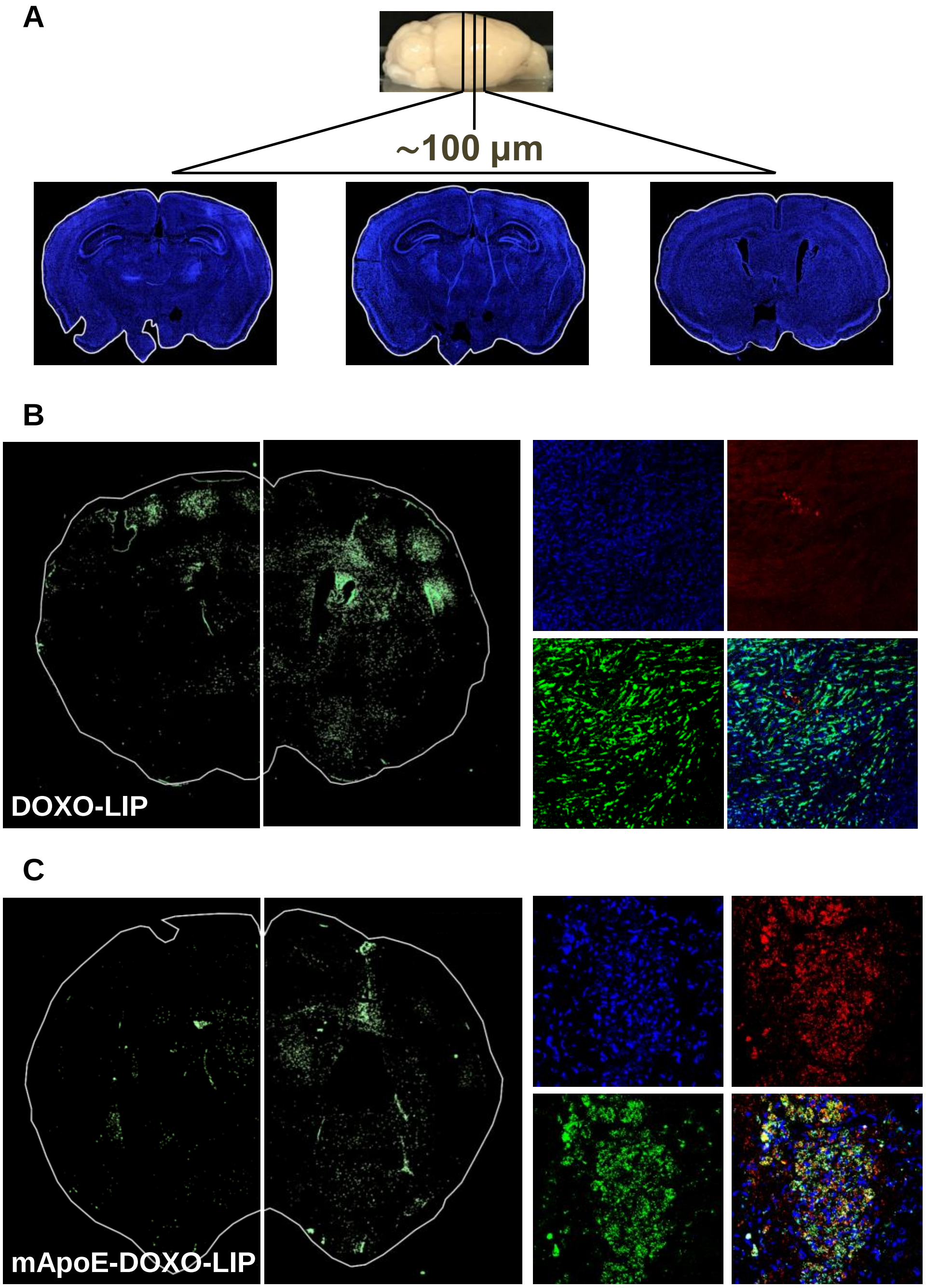

Supplementary Figure S6. Hystological analysis of tumor growth and apoptosis in GSC1luc xenografts

(A) Three coronal brain cryosections (10µm) were analyzed for each single treated and untreated mouse. Cryosections at the intersection of the tumor injection site and serial anterior/posterior sections (50 µm distance) were considered. Coronal images were acquired using a DMi8 fluorescent microscope and a Leica Application Suite X (LAS X) imaging system (Leica Microsystems). DAPI signal (blue) for nuclei detection was used to delimitate brain section contour (white lane). (B and C) Representative brain sections from DOXO-LIP and mApoE-DOXO-LIP treated mice . GSC1 cells were detected by anti-human nuclei (HU, green) immunofluorescence. Apoptotic cells were detected by byTUNEL staining (red) on the same sections. Quantification of GSC1luc cells and apoptosis are reported in Fig.5 C and D.

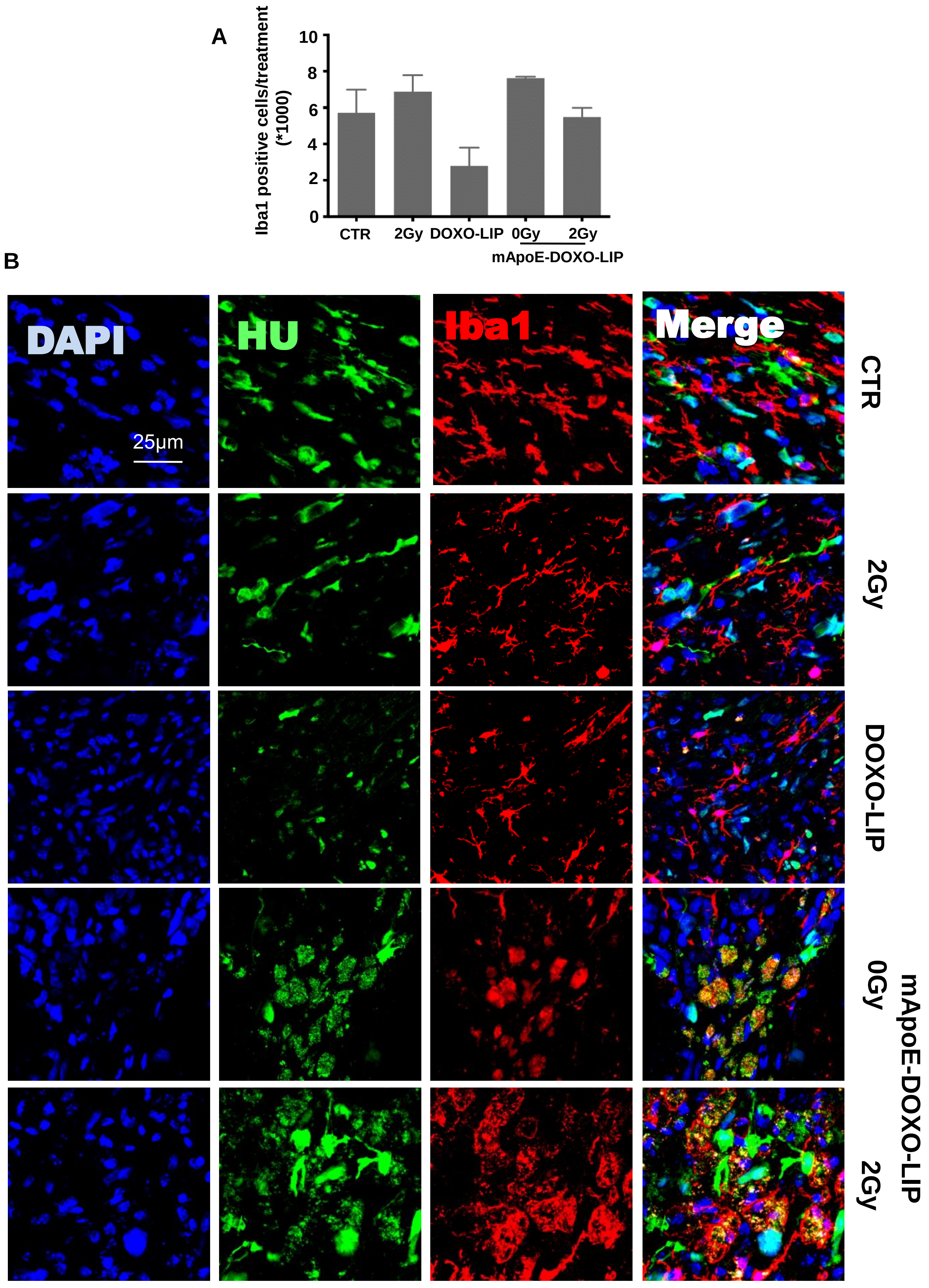
A
Iba1 positive cells/treatment (*1000)
10
8
6
4
2
0
CTR
2Gy
DOXO-LIP
0Gy
2Gy
mApoE-DOXO-LIP
B
DAPI
HU
Iba1
Merge
25µm
CTR
2Gy
DOXO-LIP
0Gy
2Gy
mApoE-DOXO-LIP

Supplementary Figure S7. Microglia/macrophage infiltration in GSC1 xenografts.

Brain cryosections were co-stained with anti-HU,anti-Iba1 and DAPI. (A) Quantification of microglia/macrophages in brain cryosections by Iba1 and DAPI staining detected using a DMi8 fluorescent microscope (Leica Microsystems). Iba1/DAPI-positive cells present in the tumor-injected hemisphere were counted in three serial section for each brain (see Supplementary Figure 5). Quantification was carried out by imaging analysis (FIJI software). Results are expressed as mean values ± SE of at least nine sections of the indicated treatment group. (B) Representative images of cryosectons in the tumoral area from untreated mice (CTR) and animals treated with radiation (2Gy), DOXO-LIPs or mApoE-DOXO-LIPs alone (0Gy) or in combination with radiation (2Gy).

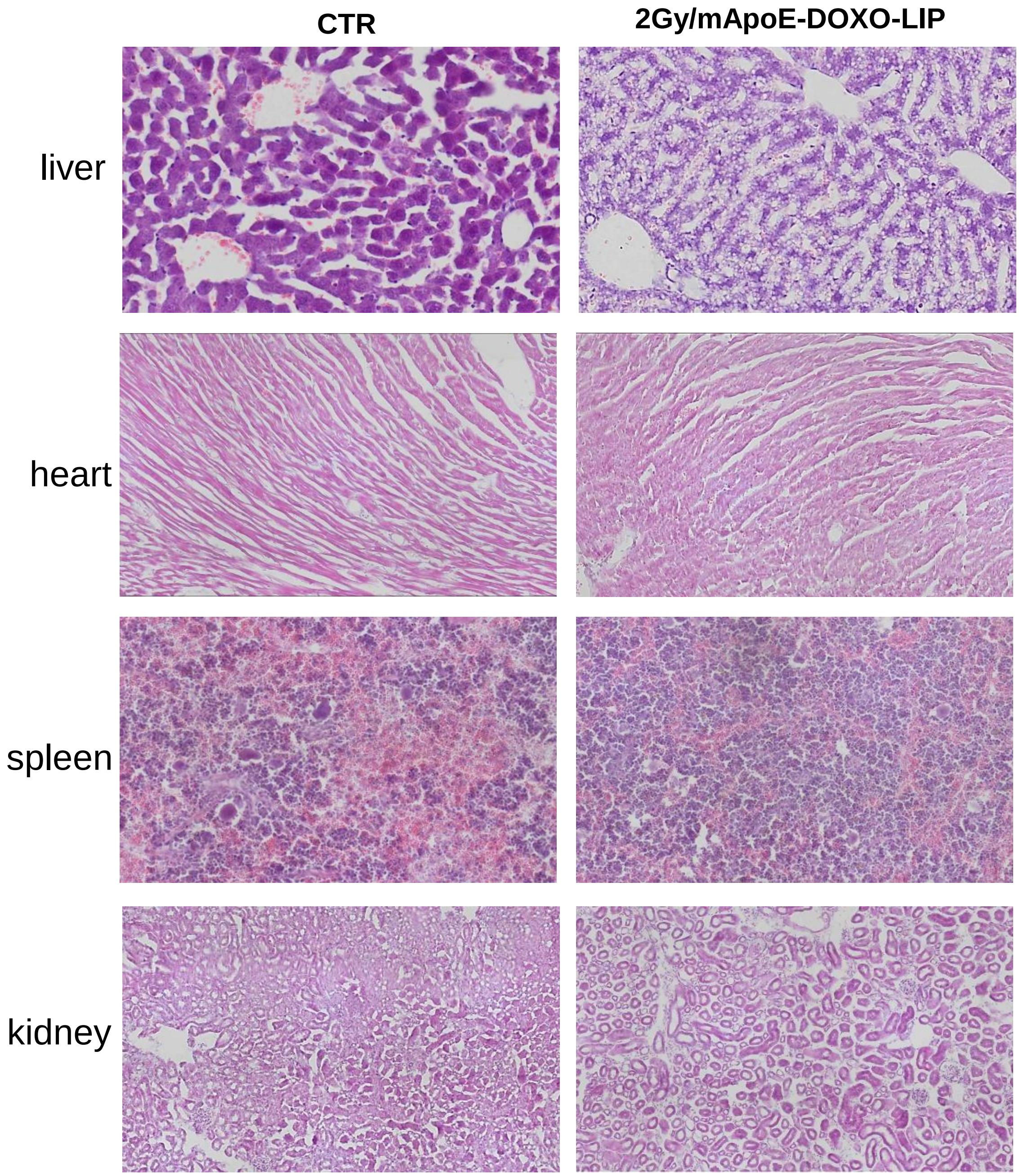

Supplementary Figure S8. Hematoxylin and eosin staining

Cryosections of peripheral organs from the same mice described in Fig. 5. Images were acquired using a VS120 dotSlide (Olympus, Tokyo, Japan).

Supplementary Methods

Preparation and characterization of mApoE-DOXO-LIPs

LIP composed of Sm/Chol/PE-PEG-mal (48.75:48.75:2.5 molar ratio) in ammonium sulphate (500 mM, pH 5.5) were prepared by extrusion procedure. LIPs (5 µmolstotal lipids) were incubated with 1 mg/ml DOXO for 1 h at 65°C to obtain DOXO-LIP. DOXO-LIPs were then functionalized with mApoE as described previously 20 to obtain mApoE-DOXO-LIPs. mApoE (CWGLRKLRKRLLR-NH2) was purchased from DBA Italia (Segrate, Italy).

LIPs were purified by gel filtration (Sephadex G-25 fine resin). DOXO loading was quantified by measuring the DOXO fluorescence (λex = 495 nm; λem =592 nm) after liposomes disruption with 0.1% Triton X-100. Phospholipids content was quantified by Stewart Assay. The amount of mApoE attached on LIP surface was determined by measuring the shift of tryptophan fluorescence intensity (λex = 280 nm). Non-functionalized LIP and DOXO-LIP were used as controls. Stability was measured in PBS by following size and polydispersity index (PDI) by DLS for 10 days. The reported data are the mean of at least five different measurements. For transmission electron microscopic (TEM) evaluation, mApoE-DOXO-LIP were deposited on formvar and carbon coated 300 mesh copper grids (EMS, Hatfield—PA) and negatively stained with a solution of 2% Uranyl Acetate. After drying, the grids were examined under a Philips CM10 TEM (FEI, Eindhoven, NL) with an accelerating voltage of 80 kV and images were acquired with a Morada camera and iTEM software (Olympus, Tokyo, JP).

In vitro DOXO release

Release of DOXO from loaded LIP was monitored at 37 °C in phosphate buffered saline (PBS) at pH 7.4, 6.5 or 5.0. At various time points up to 14 days, an aliquot was withdrawn from LIP suspension and filtered using a 10 kDa MWCO spin filter, at 4000 rpm. The filtrate, which contained DOXO released from the LIP, was analyzed for DOXO content as described above by spectrofluorimetry. DOX release was calculated using Eq. (1):

DOXO release (%) = ([DOXO]filtrate / [DOXO]initial sample) * 100

Evaluation of LIP internalization in GSCs

GSCs were seeded on glass coverslips (5x104 cells on 18mm coverslips) and incubated with DOXO-LIP or mApoE-DOXO-LIP for 4 h (DOXO, 4 µg/ml in every condition). Irradiated GSCs were incubated with LIPs 20h after radiation treatment. Cells were irradiated with a single dose of 2Gy using an X-ray biological irradiator operating at 12 mA/190kV (RADGIL, Gilardoni, Lecco, Italy) at a dose rate of 0.65 Gy/min. The Z-stack series of the cells in different experimental conditions were acquired by means of a confocal microscope (fv1000 TIRF, Olympus) with 63X1.35 OIL objective. The DAPI signal (ex/em: 405/450 nm) and DOXO autofluorescence were acquired sequentially at high scan rate. Images were processed using ImageJ software using the DAPI signal to segment the nucleus and bright field (Nomanski) to identify the cell contour. At least 30 cells/experimental group from 10 microscopy fields were analyzed.

LDLR western-blot and immunofluorescence

LDLR level of expression in GSC cultures was detected by Western blot analysis on $5x10^5$ irradiated (2Gy) and non-irradiated cells (0Gy). Mouse anti-human LDLR antibody (abcam, Cambridge, UK) was incubated 1:1000 overnight at 4°C. The signal intensity was determined by ChemiDoc Imaging System (BioRad, Hercules, CA) and analyzed by Image Lab software (BioRad, Hercules, CA). Samples were normalized on total protein amount.

For LDLR mmunostaining cells ($5x10^4$ cells on 18mm coverslips) were incubated with primary antibody against human LDLR (1:100, Sigma-Aldrich) over night at 4°C. After primary antibody, samples were incubated with anti-mouse secondary antibody conjugated with Alexa Fluor® 488 (1:200, Thermo Fisher) and anti-Phalloidin conjugated with Alexa Fluor® 647 (1:100, Thermo Fisher) for 1h at room temperature. Glasses were mounted with a PBS/glycerol mixture (1:1) with DAPI (1:40.000) (Sigma-Aldrich, St. Louis, MO). LDLR level of expression was quantified by confocal images processed using ImageJ software. The central, upper, and lower sections of 1µm z-stack were analyzed for each image and the LDLR threshold, determined on the negative control, was kept constant in each experimental conditions. LDLR level was calculated as LDLR-positive fluorescent area normalized to the cellular cytoplasmatic area demarcated by the phalloidin staining.

Supplementary Methods

In vitro viability assay

GSCs (10^4 cells/well) and endothelial hCMEC/D3 cells (5000 cells/well) were plated in 96-well plates in a final volume of 200 µl of medium in the absence/presence of increasing doses of different LIPs at the indicated doses of DOXO. Only in the experiments using the GBM integrated BBB model, LIPs were removed 3 h after treatment. 48-72 h after treatment, GSCs and/or GBM cell lines respectively, the mitochondrial enzymatic activity, index an indicator of cell viability, was assessed by MTT assay.

Histopathology analysis and TUNEL assay

Animals were euthanized with intracardiac perfusion of saline solution, followed by 4% paraformaldehyde fixation. Harvested brains were frozen, and serial cryosections (10 μm thick) were prepared and stored at -80C°. Three coronal sections (one at the intersection of the tumor injection site plus one anterior and one posterior sections at 50 μm distance(Supplementary Fig. S6) were analyzed for each single treated and untreated mouse.

Frozen brain sections were immunostained with mouse anti-Human Nuclei (clone 235-1, Sigma), rabbit anti-Iba1 (Wako), rabbit anti-eiF2α (Ser51) (Immunological Sciences), rat anti-mouse MHCII (eBioscience), rabbit anti- mouse CD11c (Cell Signaling) primary antibodies followed by anti-mouse Alexa Fluor 488, anti-rat Alexa Fluor647, anti-rabbit Alexa Fluor 555 secondary antibodies (Thermo Fisher). The Click-iT Plus Alexa Fluor 647 TUNEL Assay (Thermo Fisher) was used to detect in situ apoptosis and DAPI for nuclei staining. Coronal images were acquired using a DMi8 fluorescent microscope and Leica Application Suite X (LAS X) imaging system (Leica Microsystems). Confocal microscopy was performed using a Leica TCS SP8 confocal microscope. Maximum projections were obtained from 29-35 Z-stacks. Image analysis was carried out by FIJI (Schindelin J; Arganda-Carreras I; Frise E; et al. Fiji: an open-source platform for biological-image analysis. Nature methods 2012;9(7):676-682) or Imaris (Bitplane, Zurich, Switzerland) for 3D rendering.
